# Evaluation of different vagus nerve stimulation anatomical targets in the ear by vagus evoked potential responses

**DOI:** 10.1002/brb3.2343

**Published:** 2021-09-22

**Authors:** Iñaki Garcia de Gurtubay, Pedro Bermejo, Miguel Lopez, Iñaki Larraya, Julian Librero

**Affiliations:** ^1^ Department of Neurophysiology Complejo Hospitalario de Navarra Pamplona Spain; ^2^ Department of Neurology Puerta de Hierro Hospital Madrid Spain; ^3^ Walden Medical Neurodigital Therapies Gijón Spain; ^4^ Biomedical Research Centre of the Government of Navarre Pamplona Spain

**Keywords:** auricular branch of vagus nerve (ABVN), evoked potential, nucleus of solitary tract, transcutaneous vagus nerve stimulation, vagus sensory evoked potential (VSEP)

## Abstract

**Background:**

Electrical auricular vagus nerve stimulation (taVNS) is an emerging therapy. Stimuli are transported to brainstem nuclei, whereby its multiple projections reach to many subcortical and cortical areas, thus allowing the neuromodulation of several systemic physiological processes. We aim to define the best auricular target for taVNS through vagus somatosensory evoked potential (VSEP) elicited stimulating different auricular areas with different electrode sizes.

**Methods:**

Twenty‐six subjects were enrolled. Three stimulation areas were studied: simultaneous cymba and cavum (CC), cymba (C) and earlobe (L); and two electrode sizes: extra‐large (X) and small (S). We studied the effect of five combinations (CCX, CCS, CS, LX and LS) on VSEP´s latency and amplitude, and sensory and pain threshold (Pt) using a lineal mixed model regression analysis. We used CS combination, used in a commercial device, as reference model.

**Results:**

Valid VSEP were obtained for CCX, CCS and CS but not in LX and LS. Both CCS and CCX tests showed significant amplitude increases. The same effect was observed in CCX using CCS as reference. Significant increases in Pt were found for CCX and LX. The same effect was observed in CCX using LX as reference.

**Conclusion:**

The results suggest that CC and C areas are active targets for taVNS but not for earlobe, as anatomical data support. Considering that amplitude reflects the synchronized electrical activity generated, we conclude the most effective topography is the simultaneous stimulation of cymba and concha. The use of X‐sized electrodes increases the amplitudes and makes the stimulation more comfortable.

## Graphical abstract

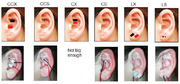

## 1 INTRODUCTION

The vagus nerve (VN) is the longest cranial nerve and is involved in the regulation of multiple systems (H. Yuan & Silberstein, 2016). Therefore, due to this influence on multiple systems and its important role in maintaining homeostasis, stimulating this nerve to modulate the function of related organs has long drawn the attention of investigators (T.‐F. Yuan et al., 2016).

As a slow‐acting therapy, cervical vagus nerve stimulation (VNS) has been approved by the US Food and Drug Administration for managing treatment refractory epilepsy in 1997 and for chronic treatment‐resistant depression in 2005 (H. Yuan & Silberstein, 2016). However, surgical risks, technical challenges and potential side effects have limited the application of VNS (Fitzgerald, 2013; Ventureyra, 2000). To overcome such barriers of applying invasive VNS (iVNS), some noninvasive transcutaneous vagus nerve stimulation (tVNS) methods have been developed, superficially stimulating the VN at the neck or at the outer ear.

The rationale of tVNS on the ear is based on anatomical studies demonstrating that certain parts of the ear area have afferent VN distribution (Henry, 2002; Peuker & Filler, 2002; Trevizol et al., 2015), and electrical stimulation of these areas may produce activity changes in the VN pathway in the brain stem and central structures (Shiozawa et al., 2014), producing a modulation effect similar to iVNS (Carreno & Frazer, 2016; Hein et al., 2013; P.‐J. Rong et al., 2012). The auricular branch of the vagus nerve (ABVN) spans from the main bundle of the VN and innervates the external ear (Berthoud & Neuhuber, 2000; Dabiri et al., 2020), although its afferent projections are still not well understood. It is known that the cymba conchae of the external ear is innervated exclusively by this branch, but other regions of the external ear receive important afferent innervation by ABVN solely (Peuker & Filler, 2002) or shared with other nerves such as the tragus, the posterior and inferior walls of the ear canal (Fay, 1927; Tekdemir et al., 1998) and the cavity of the conchae (Berthoud & Neuhuber, 2000). So, using ABVN as target of noninvasive brain stimulation is known as transcutaneous auricular vagus nerve stimulation (taVNS). taVNS has been used to treat disorders, such as epilepsy (P. Rong et al., 2014; Stefan et al., 2012), prediabetes (Huang et al., 2014), depression (H. Yuan & Silberstein, 2016), chronic tinnitus (Shim et al., 2015), migraine (Silberstein et al., 2016), rehabilitation after ischemic stroke (Baig et al., 2019), ventricular arrhythmias (Nasi‐Er et al., 2019), respiratory symptoms associated to COVID‐19 (Staats et al., 2020) as well as to boost associative memory (Jacobs et al., 2015) what has been proposed to help patients with Alzheimer's disease and other dementia types (Cai et al., 2019; Kaczmarczyk et al., 2017).

Those clinical effects depend on various parameters of the taVNS such as the electrode position, stimulus duration, interstimulus interval and the stimulus intensity (Hagen et al., 2014). However, currently different stimulation targets and parameters are used, which make it difficult to evaluate and compare the potential therapeutic applications.

Investigators have recently focused the discussion on which anatomical target is most biologically active. The vagally innervated region of the ear is usually selected to stimulate according to sparse and limited human auricle dissection anatomical studies (Bermejo et al., 2017; Oken, 1997) that point the cymba conchae and cavum, as well as the external auditory channel as the highest density areas of ABVN projections. According to these findings, the wider commercialized medical taVNS device (NEMOS; Cerbomed, Germany) stimulates over cymba conchae. On the other hand, due to the controversy on the best anatomical location for stimulation and whether the stimulation effects of taVNS are due to the recruitment of ABVN or other nerves, different targets have been proposed (Kaniusas, Tittgemeyer, Panetsos, Gines, Papa, Kiss, Podesser, Cassara, Tanghe, Samoudi, Tarnaud, Joseph, Marozas, Lukosevicius, Ištuk, Šarolić, et al., 2019) such as cymba conchae or cavum (Ay et al., 2015; Suk et al., 2018), the tragus (Badran et al., 2018), the external auditory channel or some of its specific parts (Skraus et al., 2013).

Fallgatter et al. (2003) developed a noninvasive method for the measurement of vagus nerve function by evoked potentials (EP) recorded after electrical stimulation of ABVN. Cutaneous stimuli are transported via the auricular nerve to the jugular ganglion and from there with the vagus nerve into the medulla oblongata and to the nuclei tractus solitarii (NTS). This nuclear region of the vagus nerve has multiple projections to many subcortical and cortical brain regions, which explain the long‐distance physiological modulation of vagus stimulation. The postsynaptic brainstem activity from the VN nuclei can be recorded at the scalp as far field potential (Fallgatter et al., 2003; Polak et al., 2009; Usami et al., 2013) called vagus somatosensory evoked potential (VSEP) and is only elicited when stimulating within the innervation area of the ABVN, while no evoked potential will be recorded stimulating at other sites. The characteristics of this VSEP have been described in normal population (Fallgatter et al., 2003; Lewine et al., 2019; Polak et al., 2009) as well as pathological conditions (Polak et al., 2017, 2013, 2014, 2007, 2011). On the other hand, VSEP obtained by taVNS are like those seen with invasive VNS (Nonis et al., 2017).

The amplitude of an evoked potential mainly reflects the amount of electric potentials generated along the stimulated neural pathway as a representation of the amount of stimulated neural fibers. So, the aim of our study was to define the best auricular target for taVNS comparing the characteristics of VSEP elicited when stimulating over three different auricular sites with two electrode sizes and to define the optimal combination that evokes higher amplitude VSEP. As secondary objective, we wanted to find optimal stimulation intensity ranges, sufficient and acceptable to generate VSEP but not reaching to be painful, that could be used for prolonged in time taVNS clinical studies.

## 2 MATERIALS AND METHODS

A total of 26 healthy volunteers participated in the present study, approved by the ethics committee of our centre. Written informed consent was obtained from all participants. The exclusion criteria were (1) intake of any medication, (2) history of neurosurgical treatment, (3) ear lesions or infections, (4) allergy to Ag/AgCl, (5) pregnancy or breastfeeding, (6) history of neuropathy (including autonomic or diabetic neuropathy) and (7) history of traumatic brain injury.

After careful preparation of the skin with Nuprep abrasive paste, two Ag/AgCl (Ambu Neuroline 726 20 M/10) electrodes were fixed to the scalp with Ten 20 conductive paste in F3 and C3 positions of the international 10–20 system. A third electrode was placed in top of the left shoulder and used as ground. Electrode impedance was always below < 2kΩ and checked before and after the stimulating period of every test.

To obtain the stimulation electrodes, we removed the external PVC surface and the solid gel adhesive from commercial Ambu Neuroline 715 electrodes. These electrodes are flexible in nature and reasonably guarantee an adequate fitting with the normal skin tissue irregularities of ear surface. Then, we obtained a 5.2 × 10.4 mm (54 mm^2^) Ag/AgCL electrode that we call extra‐large electrode (X). To obtain the small electrode (S), we cut and reduced the surface of X electrode to 2.3 × 2.3 mm (5.3 mm^2^). The size of S electrode was chosen to be similar to the stimulation surface used by the NEMOS‐Cerbomed device, one of the most widely used commercial taVNS systems (Burger et al., 2018; Frangos et al., 2015).

**FIGURE 1:**
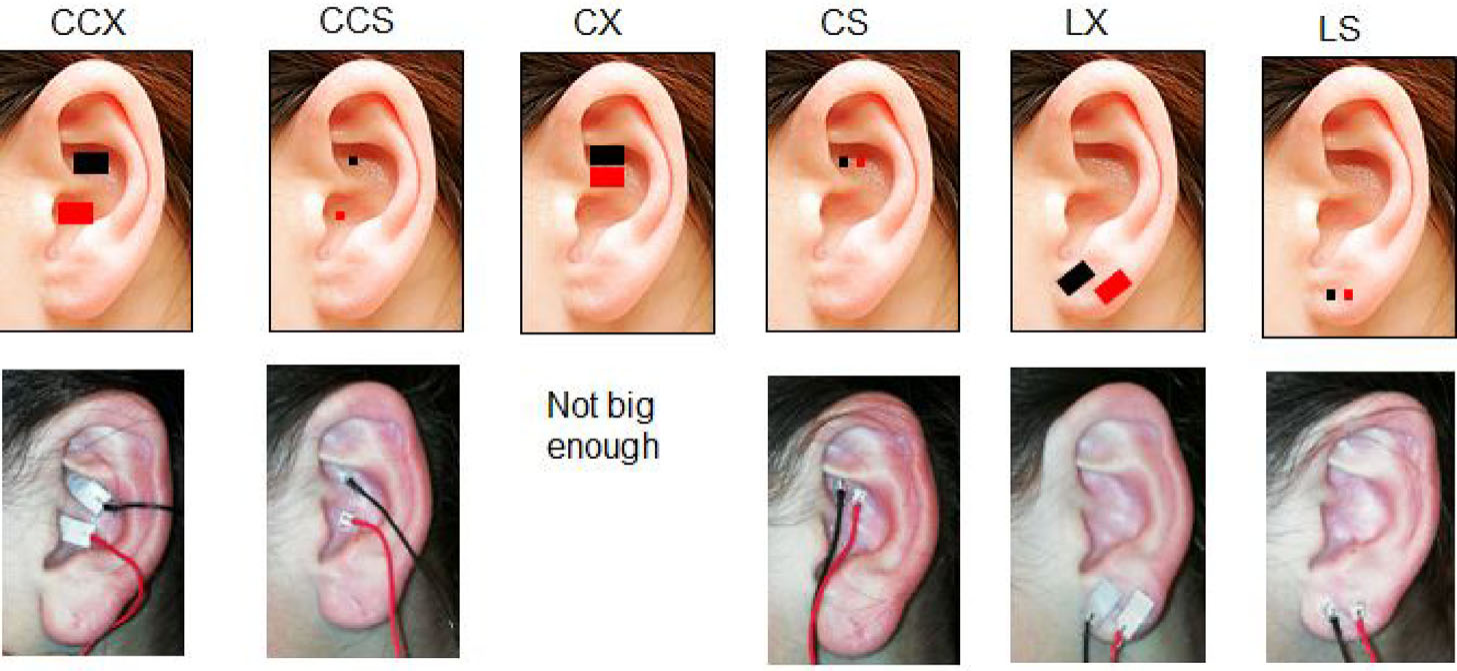
Stimulated areas: Topography and electrode size combination. Topography: CC cymba and cavum, C cymba, L lobe. Electrode size: X extralarge, S small

We selected three different stimulation topographies, all of them in the left ear: cymba and cavum (CC) with cathode in cymba and anode in cavum conchae, cymba (C) with cathode and anode with 3 mm interelectrode distance; and lobe (L) with both electrodes over the ear lobe. We cleaned the ear with alcohol, dried it and used Collodion SLE‐UK medical adhesive to attach stimulating electrodes over these areas. So, from the combination of three topographies and two electrode sizes, we conducted five different experimental paradigms called tests to our volunteers in a single afternoon session: CCX, CCS, CS, LX and LS. CX test was not done because there was not enough space to attach X size electrode over the cymba (Figure 1).

Electrical brain activity was recorded and data were processed with a commercial EMG and evoked potential system (Synergy Viasys HealthCare) and software V.15.0).

We used the same electrical stimulation parameters for the five tests, with electrical 500 μs square impulses of alternating polarity and 1 Hz frequency. At the beginning of each test, the sensory threshold (St) was measured with a progressive increase of stimulation intensity from 0 mA to that at which the subject began to perceive the stimuli. Then, we also measured the pain threshold (Pt), the lowest intensity at which stimuli were perceived as painful. To avoid stimulation of other nerve fibers or artefact responses, we never stimulated over 5 mA.

For each subject, the first test was always CS, and the remaining tests were randomly assigned. After measuring St and Pt in CS condition, we calculated the stimulus intensity value that we used to elicit the VSEP increasing by 2.5 the St value. We fixed this intensity also for the remaining test regardless their specific St value. Then, we asked volunteers to relax themselves and to close their eyes and we began the test.

VSEP were recorded over the F3‐C3 bipolar derivation (Hagen et al., 2014). To avoid contaminated responses and consider a VSEP as valid, at least 50 consecutive artifact free signals must be averaged with the automatic artifact rejection system at ±30 μV. When we obtained at least two stable and consistent valid VSEP, we continued to the next test. The parameters of the VSEP manually measured for every condition were latency to the VSEP onset, onset to peak amplitude and duration. The experimenters evaluating the study outcomes were blinded to the order of the stimulation parameters.

We proceeded to investigate the effect of different topography and size combination on latency, amplitude, sensory threshold and pain threshold, using a lineal mixed model regression analysis with topography and size as fixed effects and individuals as random intercept. The intercept is the mean for the reference group (CS combination). We choose a sample size that allows to process it as normally distributed (Oken, 1997). All data were preprocessed and statistically analyzed using the R software package Ime4.

## 3 RESULTS

### 3.1 Timing of the procedure

The timing of the procedure for any test was as follow: 5 min to prepare skin, reduce and measure impedances, fix stimulating electrodes, and let the adhesive dry. Then, 2 min to calculate thresholds (applying intermittent stimulation), followed by 4 to 8 min of electric stimulation (stimulation period). It was administered in several series of continuous stimulation (1 min 15 sec) separated by rest periods (15 sec) until two valid VSEP were obtained. Rest periods were used to decide if obtained VSEP was accepted or rejected. Finally, 2 min were used to remove stimulating electrodes and clean the skin. Then, the procedure started again for the following test. With this procedure, the mean time consumption for a test was of around 15 min (range 13–17). The time between the stimulation periods of two different tests was 9 min, and the time for the complete session was 1 h and 15–25 min.

### 3.2 Descriptive data

**FIGURE 2:**
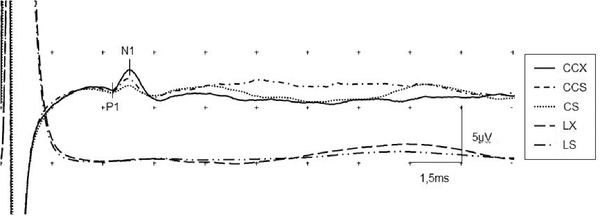
Evoked potentials at electrode position C3‐F3 for a single subject. Stimulation intensity 2.2 mA. Amplitude P1‐N1 values at five different topography and electrode size combinations: CCX 2.45 μV; CCS 1.45 μV; CS 0.65 μV; LX and LS not measurable

**FIGURE 3:**
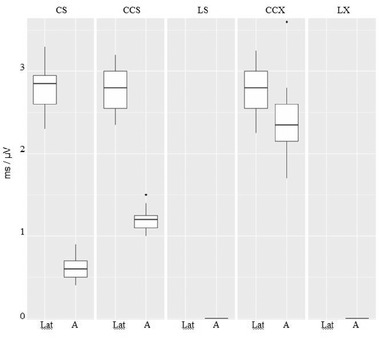
Latency (lat) and amplitude (a) values for topography and electrode size combination on CS, CCS, LS, CCX, LX test. Mean values are represented as a horizontal lines, with standard deviation (box), range (vertical line) and outliers (point)

**FIGURE 4:**
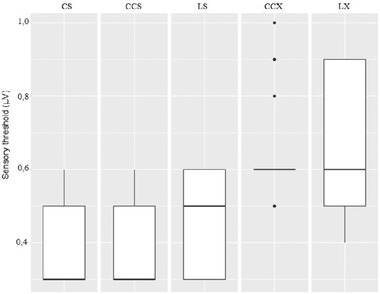
Sensory threshold group values for CS, CCS, LS, CCX and LX topography and electrode size combinations: Mean group value is represented as a horizontal line, with standard deviation (box), range (vertical line) and outliers (point)

**FIGURE 5:**
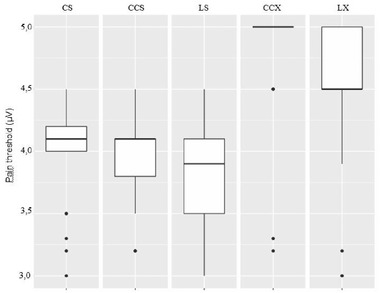
Pain threshold (Pt) group values. Stimulating with S electrode, in all conditions all subjects fixed the pain threshold in values under 5 mA. Otherwise, stimulating with X electrode 20 subject in CCX test and 7 in CC test reached to our previously fixed as maximum stimulation intensity of 5 mA with no pain sensation at all

We obtained valid VSEP in 25 individuals (12 women, 13 men) from 22 to 59 years old. A male subject did not reach enough relaxation to get an acceptable baseline to obtain VSEP and was rejected. No adverse effects were observed in the study. Evoked responses drawing for a single subject are shown in Figure 2. In the CS test, all subjects showed a clear VSEP, with mean group latency 2.79 ± 0.28 ms and mean group amplitude 0.61 ± 0.13 μV. Mean group St was 0.41 ± 0.13 mA y mean group Pt was 3.94 ± 0.40 mA with no subject reaching to 5 mA. The mean stimulation intensity used to obtain VSEP was 2.4 mA. In the CCS test, all subjects showed a clear VSEP with mean group latency 2.78 ± 0.26 ms and mean group amplitude 1.21 ± 0.13 μV. Mean group St was 0.39 ± 0.10 mA and mean group Pt was 3.89 ± 0.36 mA with no subject reaching to 5 mA. In the LS test, no VSEP was obtained for any subject of the group. Mean group St was 0.46 ± 0.12 mA, and mean group Pt was 3.78 ± 0.43 mA with no subject reaching to 5 mA. In the CCX test, all subjects showed a clear VSEP with mean group latency 2.77 ± 0.28 ms and mean group amplitude 2.39 ± 0.38 μV. Mean group St was 0.66 ± 0.14 mA and mean group Pt was 4.8 ± 0.49 mA. During the measurement of Pt, 20 individuals reached to 5 mA without complaining about pain or discomfort. In the LX test, no VSEP was obtained for any subject of the group. Mean group St was 0.64 ± 0.19 mA and mean group Pt was 4.47 ± 0.51 mA. During the measurement of Pt, seven individuals reached to 5 mA without complaining about pain or discomfort. Mean group data values are shown in Figure 3 for latency and amplitudes, Figure 4 for St and Figure 5 for Pt.

### 3.3 Comparisons of latencies

No statistical differences were found between the latencies of our reference test (CS) and CCS (*p* = .303) or CCX (*p* = .094) tests (Table 1a).

**TABLE 1:** Statistical comparison (lineal mixed model regression analysis) between experimental combinations. Intercept is the test used as reference. The mean value for intercept is shown in estimates column. The remaining of values in that column show proportional increases (positive) or decreases (negative) with respect to the reference

A	Lat			A			St			Pt		
Predictors	Estimates	CI	*p*‐Value	Estimates	CI	*p*‐Value	Estimates	CI	*p*‐Value	Estimates	CI	*p*‐Value
Intercept (CS)	2.79	2.68–2.90	<.001	0.61	0.51–0.70	<.001	0.41	0.35–0.46	<.001	3.94	3.76–4.11	<.001
CCS	−0.02	−0.05 to 0.01	.303	0.61	0.48–0.74	<.001	−0.02	−0.07 to 0.04	.567	‐0.04	−0.21–0.12	.597
CCX	−0.03	−0.06 to 0.00	.094	1.79	1.66–1.92	<.001	0.26	0.20–0.31	<.001	0.86	0.70–1.03	<.001
LS							0.06	0.00–0.11	.045	‐0.15	−0.32 to 0.01	0.068
LX							0.23	0.18–0.29	<.001	0.54	0.37–0.70	<.001
Random effects												
δ^2^	0.00			0.05			0.01			0.09		
τ_00_	0.07_id_			0.01 _id_			0.01 _id_			0.11 _id_		
ICC	0.96			0.11			0.51			0.56		
N	25 _id_			25 _id_			25 _id_			25 _id_		
Observations	75			75			125			125		
Marginal R2/conditional R2	0.002 / 0960			0.904/0.915			0.406/0.707			0.440/0754		
												
	b	A					C		Pt			
		Predictors	Estimates	CI	*p*‐Value				Predictors	Estimates	CI	*p*‐Value
	Intercept (CCS)		1.21	1.12–1.31	<.001		Intercept (LX)			4.47	4.30–4.65	<.001
	CCX		1.18	1.05–1.31	<.001		CCX			0.33	0.16–0.49	<.001

Abbreviations: A, amplitude; CI, 95% confidence interval; CS, cymba small; CCS, cymba and cavum small; CCX, cymba and cavum extra‐large; ICC, intraclass correlation coefficient; LS, lobe small; LX, lobe extra‐large; Lat, latency; Pt, pain threshold; St, sensory threshold.

### 3.4 Comparisons of amplitudes

When analyzing the amplitudes of the VSEP and using CS combination as reference values for them, we found significant (*p* < .001) increases 1.79 (CI 95% 1.66‐1.92) in CCX test and significant (*p* < .001) higher amplitudes 0.61 (CI 95% 0.48–0.74) in CCS combination. This last comparison avoids the influence of electrode size (Table 1a).

To avoid the influence of topography, we reanalyzed and using CCS data as reference we compared CCX test amplitudes (Table 1b). We found significant increases (*p* < .001) in the amplitudes 1.18 (CI 95% 1.05–1.31) for this last test (Table 1b).

### 3.5 Comparisons of sensory threshold

Compared to the CS combination, the sensory threshold for CCS did not produce any significant differences (*p* = .567). The slightly higher mean threshold values obtained for remaining tests were significant for LS (*p* = .045), CCX (*p* < .001) and LX (*p* < .001) as shown in Table 1a.

### 3.6 Comparisons of pain threshold

Compared to the CS combination, the pain threshold for the tests using S size electrodes were not significant (*p* = .597 for CCS and *p* = .068 for LS). However, the increases of Pt registered for CCX 0.86 (CI 95% 0.70–1.03) and for LX 0.54 (CI 95% 0.37–0.70) resulted both significantly higher (*p* < .001) as shown in Table 1a.

Comparing with LX test, we found that the Pt increases in CCX test 0.33 (CI 95% 0.16–0.49) were significant (*p* < .001) as shown in Table 1c.

## 4 DISCUSSION

To the best of our knowledge, this is the first study comparing the classical cymba conchae stimulation and the cavum and cymba conchae simultaneous stimulation. Our findings reveal that stimulation over C and CC topographies in healthy adults produces clear and reproducible evoked potential with latency and morphology close to those described previously as VSEP (Fallgatter et al., 2003) which reflects far field potentials of postsynaptic brainstem activity from the VN nuclei that can be elicited on electrical stimulation of the ABVN (Fallgatter et al., 2003; Polak et al., 2009). In contrast, these evoked potentials are not registered when we stimulate over the earlobe. This suggests that stimulating over C and CC topographies activates vagus pathway but it is not activated when stimulating over the earlobe. Assuming that the amplitude of an EP reflects the amount of synchronized electrical activity generated as a consequence of the stimulation, as we systematically obtained significant higher responses on CC topography, being independent of which electrode size has been used to stimulate, we could conclude that the stimulation of the ABVN is also higher. This suggests that cavum is a likely biologically active target, able to stimulate the ABVN and to enhance the classically used cymba conchae stimulation. On the other hand, our finding points that the earlobe is not an active target, at least with respect to ABVN stimulation.

Anatomical data support the use of these two locations, cymba and cavum conchae, for taVNS. Cymba conchae has clearly a large number of VN fiber endings with 100% of ABVN nerve supply (Peuker & Filler, 2002), and its stimulation has demonstrated to produce significant activation of the classical central vagal nerve projections, for example, widespread activity in the ipsilateral NTS, bilateral spinal trigeminal nucleus, dorsal raphe, locus coeruleus, contralateral parabrachial area, amygdala and nucleus accumbens (Badran et al., 2017; Frangos et al., 2015; Yakunina et al., 2017). Some medical devices (e.g., NEMOS, Cerbomed GmbH) have been commercialized to stimulate this area and treat different neurological and psychiatric disorders. On the other hand, an important VN innervation of cavum conchae and external auditory channel has been recently described by our group (Bermejo et al., 2017) with a variable percentage of myelinated nerve fibers, therefore able to be activated by transcutaneous stimulation. These results are supported by other previous anatomical data (Ellrich, 2019; Safi et al., 2016) that demonstrate the existence and quantity of thick‐myelinated afferent nerve fibers of the left auricular branch of the VN that carries a variable percentage of thick‐myelinated afferent nerve fibers counted in the left thoracic VN in humans. So, that is why cavum has already been considered as a potential target to be used in taVNS therapies. Suk et al. (2018) suggested that isolated stimulation of cavum was more effective than cymba stimulation for the treatment of tinnitus. Ay et al. (2015) proposed the stimulation of this structure as a target for the protection of ischemic stroke in rats.

Most of these cavum and cymba conchae stimuli are driven by ABVN (He et al., 2013), forming the so‐called auriculo‐vagal pathway (Ginsberg & Eicher, 2000), but other nerves involved in the afferent innervation of the auricular area drive afferent signals of different nature to NTS. In fact, with regard to the anatomical perspective, the cavum is innervated by both the ABVN and the greater auricular nerve (GAN) with 45% and 55% nerve supply, respectively (Peuker & Filler, 2002). Simultaneous stimulation over cymba and cavum regions of the ear increases the amount of activated ABVN fibers than cymba conchae stimulation alone, but makes it difficult to link the results to a specific neural pathway because of the dual innervation of cavum. Pragmatically, the objective during atVNS is to depolarize the NTS as first step to activate the VN pathway in the brain stem and central structures to obtain modulatory effects. The NTS and associated tract receive afferents via the facial, glossopharyngeal and vagus nerves. GAN is the superficial branch of the cervical plexus from the C2 and C3 spinal nerves and is divided into anterior and posterior branches. GAN does not project fibers directly over NTS, but the posterior branch communicates with ABVN and the posterior auricular branch of the facial nerve (Ginsberg & Eicher, 2000). As registered evoked potential reflects the postsynaptic brainstem activity originated in NTS, we cannot discard some influence of the stimulated GAN fiber of cavum on the VSEP results, but we believe that is unlikely. Such an indirect long neural pathway would arrive later than direct afferents to NTS, producing a temporal dispersion of the VSEP and consequently a reduction in its amplitude. On the other hand, the ear lobule, which has been frequently used as a sham stimulation site in many taVNS studies, is innervated by GAN alone and does not generate VSEP at all. So, the anatomical distribution supports our results of obtaining an objective and measurable VSEP response when stimulating over C or CC areas, as a result of well synchronized postsynaptic potentials from jugular ganglion and NTS, but not over the earlobe.

Therefore, although the effectiveness of the NTS stimulation might vary in relation to the nerve stimulated, it seems evident that the stimulation of different auricular areas can activate NTS or other brainstem structures. Thus, the term auricular stimulation or neuromodulation could be more correct than the term taVNS (Mercante et al., 2018). This way, the global effects of auricular transcutaneous nerve stimulation results in activation of cerebral centers other than NTS, and presumably all together elaborates the response to the stimulation. Additionally, various communications of different nerve branches around the external acoustic meatus and the auricle before reaching the central nervous system have been described (Kiyokawa et al., 2014) what contributes to an individualized and specific response. According to previous data, a new approach to taVNS considers the concomitant stimulation of auricular (Kaniusas, Tittgemeyer, Panetsos, Gines, Papa, Kiss, Podesser, Cassara, Tanghe, Samoudi, Tarnaud, Joseph, Marozas, Lukosevicius, Ištuk, Šarolić, et al., 2019; Usami et al., 2013; Yakunina et al., 2017) or even extra‐auricular (Deuchars et al., 2018) nerves in addition to the VN.

With respect to stimulating electrode size and for a given stimulation strength of St x2.5 mA, our experiment showed greater VSEP amplitudes when using X size electrodes compared to smaller ones, being independent of which topography has been stimulated, suggesting a better recruitment of ABVN fibers close to the stimulation area.

Moreover, the tests that used X size electrodes systematically showed higher Pt over the same topographic targets, allowing the distribution of the electrical charge over a greater surface, and decreasing the intensity of the charge. Using greater electrodes over confirmed topographic targets allows to improve the stimulation efficiency to reach adequate therapeutic effects with lower intensities, specially avoiding the over‐stimulation, and at the same time being more comfortable for the subject. This evidence is extremely important because therapeutically taVNS is usually applied in several daily sessions lasting from minutes to hours (Kaniusas, Tittgemeyer, Panetsos, Gines, Papa, Kiss, Podesser, Cassara, Tanghe, Samoudi, Tarnaud, Joseph, Marozas, Lukosevicius, Ištuk, Lechner, et al., 2019). The adverse effects due to stimulation parameters are mainly associated with the use of intensities over 10 mA and frequencies over 50 Hz, that have to be avoided (Hagen et al., 2014; Kaniusas, Kampusch, Tittgemeyer, Panetsos, Gines, Papa, Kiss, Podesser, Cassara, Tanghe, Samoudi, Tarnaud, Joseph, Marozas, Lukosevicius, Ištuk, Šarolić, et al., 2019; Kaniusas, Kampusch, Tittgemeyer, Panetsos, Gines, Papa, Kiss, Podesser, Cassara, Tanghe, Samoudi, Tarnaud, Joseph, Marozas, Lukosevicius, Ištuk, Lechner, et al., 2019).

To sum up, we can conclude that from the tested combinations, we have identified two easily optimizable parameters for taVNS devices that elicit higher VSEP. On the one hand, performing a simultaneous stimulation over cymba and cavum regions of the ear would produce better response than cymba conchae stimulation alone. On the other hand, the use of X‐sized electrodes improves vagus pathway activation and makes more comfortable the perception of the electrical stimuli. Both hypothetically could be related with a more intense clinical response and should be addressed in clinical trials.

### PEER REVIEW

The peer review history for this article is available at https://publons.com/publon/10.1002/brb3.2343

## Data Availability

All data generated or analyzed during this study are included in this published article.

## References

[brb32343-bib-0001] Ay, I. , Napadow, V. , & Ay, H. (2015). Electrical stimulation of the vagus nerve dermatome in the external ear is protective in rat cerebral ischemia. Brain Stimulation, 8, 7–12. 10.1016/j.brs.2014.09.009 25312600 PMC4277719

[brb32343-bib-0002] Badran, B. W. , Dowdle, L. T. , Mithoefer, O. J. , Labate, N. T. , Coatsworth, J. , Brown, J. C. , Devries, W. H. , Austelle, C. W. , Mcteague, L. M. , & George, M. S. (2018). Neurophysiologic effects of transcutaneous auricular vagus nerve stimulation (taVNS) via electrical stimulation of the tragus: A concurrent taVNS/fMRI study and review. Brain Stimulation, 11, 492–500. 10.1016/j.brs.2017.12.009 29361441 PMC6487660

[brb32343-bib-0003] Badran, B. W. , Glusman, C. E. , Badran, A. W. , Austelle, C. W. , Devries, W. H. , Borckhardt, J. J. , & George, M. S. (2017). The physiological and neurobiological effects of transcutaneous auricular vagus nerve stimulation (taVNS). Brain Stimulation, 10, 378. 10.1016/j.brs.2017.01.118

[brb32343-bib-0004] Baig, S. S. , Falidas, K. , Laud, P. J. , Snowdon, N. , Farooq, M. U. , Ali, A. , Majid, A. , & Redgrave, J. N. (2019). Transcutaneous auricular vagus nerve stimulation with upper limb repetitive task practice may improve sensory recovery in chronic stroke. Journal of Stroke Cerebrovascular Diseases, 28, 104348. 10.1016/j.jstrokecerebrovasdis.2019.104348 31570261

[brb32343-bib-0005] Bermejo, P. E. , López, M. , Larraya, I. , Chamorro, J. , Cobo, J. L. , Ordoñez, S. , & Vega, J. A. , (2017). Innervation of the human cavum conchae and auditory canal: Anatomical basis for transcutaneous auricular nerve stimulation. BioMed Research International, 2017, 7830919. 28396871 10.1155/2017/7830919 PMC5371220

[brb32343-bib-0006] Berthoud, H.‐R. , & Neuhuber, W. L. (2000). Functional and chemical anatomy of the afferent vagal system. Autonomic Neuroscience, 85, 1–17. 10.1016/S1566-0702(00)00215-0 11189015

[brb32343-bib-0007] Burger, A. M. , Van Diest, I. , Van Der Does, W. , Hysaj, M. , Thayer, J. F. , Brosschot, J. F. , & Verkuil, B. (2018). Transcutaneous vagus nerve stimulation and extinction of prepared fear: A conceptual non‐replication. Scientific Reports, 8, 11471. 10.1038/s41598-018-29561-w 30065275 PMC6068181

[brb32343-bib-0008] Cai, L. , Lu, K. , Chen, X. , Huang, J.‐Y. , Zhang, B.‐P. , & Zhang, H. (2019). Auricular vagus nerve stimulation protects against postoperative cognitive dysfunction by attenuating neuroinflammation and neurodegeneration in aged rats. Neuroscience Letters, 703, 104–110. 10.1016/j.neulet.2019.03.034 30904576

[brb32343-bib-0009] Carreno, F. R. , & Frazer, A. (2016). The allure of transcutaneous vagus nerve stimulation as a novel therapeutic modality. Biological Psychiatry, 79, 260–261. 10.1016/j.biopsych.2015.11.016 26796874

[brb32343-bib-0010] Dabiri, B. , Kampusch, S. , Geyer, S. H. , Le, V. H. , Weninger, W. J. , Széles, J. C. , & Kaniusas, E. (2020). High‐resolution episcopic imaging for visualization of dermal arteries and nerves of the auricular cymba conchae in humans. Frontiers in Neuroanatomy, 14, 22. 10.3389/fnana.2020.00022 32477074 PMC7236887

[brb32343-bib-0011] Deuchars, S. A. , Lall, V. K. , Clancy, J. , Mahadi, M. , Murray, A. , Peers, L. , & Deuchars, J. (2018). Mechanisms underpinning sympathetic nervous activity and its modulation using transcutaneous vagus nerve stimulation. Experimental Physiology, 103, 326–331. 10.1113/EP086433 29205954 PMC5887928

[brb32343-bib-0012] Ellrich, J. (2019). Transcutaneous auricular vagus nerve stimulation. Journal of Clinical Neurophysiology, 36, 437–442. 10.1097/WNP.0000000000000576 31688327

[brb32343-bib-0013] Fallgatter, A. J. , Neuhauser, B. , Herrmann, M. J. , Ehlis, A. ‐ C. , Wagener, A. , Scheuerpflug, P. , Reiners, K. , & Riederer, P. (2003). Far field potentials from the brain stem after transcutaneous vagus nerve stimulation. Journal of Neural Transmission, 110, 1437–1443. 10.1007/s00702-003-0087-6 14666414

[brb32343-bib-0014] Fay, T. (1927). Observations and results from intracranial section of glossopharyngeus and vagus nerves in man. Journal of Neurology and Psychopathology, 8, 110–123. 10.1136/jnnp.s1-8.30.110 PMC1068522 21611246

[brb32343-bib-0015] Fitzgerald, P. B. (2013). Non‐pharmacological biological treatment approaches to difficult‐to‐treat depression. Medical Journal of Australia, 199, 48–51. 10.5694/mja12.10509 25370288

[brb32343-bib-0016] Frangos, E. , Ellrich, J. , & Komisaruk, B. R. (2015). Non‐invasive access to the vagus nerve central projections via electrical stimulation of the external ear: fMRI Evidence in Humans. Brain Stimulation, 8, 624–636. 10.1016/j.brs.2014.11.018 25573069 PMC4458242

[brb32343-bib-0017] Ginsberg, L. E. , & Eicher, S. A. (2000). Great auricular nerve: Anatomy and imaging in a case of perineural tumor spread. American Journal of Neuroradiology, 21, 568–571. 10730653 PMC8174985

[brb32343-bib-0018] Hagen, K. , Ehlis, A.‐C. , Schneider, S. , Haeussinger, F. B. , Fallgatter, A. J. , & Metzger, F. G. (2014). Influence of different stimulation parameters on the somatosensory evoked potentials of the nervus vagus—How varied stimulation parameters affect VSEP. Journal of Clinical Neurophysiology, 31, 143–148. 10.1097/WNP.0000000000000038 24691232

[brb32343-bib-0019] He, W. , Jing, X.‐H. , Zhu, B. , Zhu, X.‐L. , Li, L. , Bai, W.‐Z. , & Ben, H. (2013). The auriculo‐vagal afferent pathway and its role in seizure suppression in rats. BMC Neuroscience, 14, 85. 10.1186/1471-2202-14-85 23927528 PMC3751281

[brb32343-bib-0020] Hein, E. , Nowak, M. , Kiess, O. , Biermann, T. , Bayerlein, K. , Kornhuber, J. , & Kraus, T. (2013). Auricular transcutaneous electrical nerve stimulation in depressed patients: A randomized controlled pilot study. Journal of Neural Transmission, 120, 821–827. 10.1007/s00702-012-0908-6 23117749

[brb32343-bib-0021] Henry, T. R. (2002). Therapeutic mechanisms of vagus nerve stimulation. Neurology, 59, S3–S14. 10.1212/WNL.59.6_suppl_4.S3 12270962

[brb32343-bib-0022] Huang, F. , Dong, J. , Kong, J. , Wang, H. , Meng, H. , Spaeth, R. B. , Camhi, S. , Liao, X. , Li, X. , Zhai, X. , Li, S. , Zhu, B. , & Rong, P. (2014). Effect of transcutaneous auricular vagus nerve stimulation on impaired glucose tolerance: A pilot randomized study. BMC Complementary and Alternative Medicine, 14, 203. 10.1186/1472-6882-14-203 24968966 PMC4227038

[brb32343-bib-0023] Jacobs, H. I. L. , Riphagen, J. M. , Razat, C. M. , Wiese, S. , & Sack, A. T. (2015). Transcutaneous vagus nerve stimulation boosts associative memory in older individuals. Neurobiology of Aging, 36, 1860–1867. 10.1016/j.neurobiolaging.2015.02.023 25805212

[brb32343-bib-0024] Kaczmarczyk, R. , Tejera, D. , Simon, B. J. , & Heneka, M. T. (2017). Microglia modulation through external vagus nerve stimulation in a murine model of Alzheimer's disease. Journal of Neurochemistry, 146(1), 76–85. 10.1111/jnc.14284 29266221

[brb32343-bib-0025] Kaniusas, E. , Kampusch, S. , Tittgemeyer, M. , Panetsos, F. , Gines, R. F. , Papa, M. , Kiss, A. , Podesser, B. , Cassara, A. M. , Tanghe, E. , Samoudi, A. M. , Tarnaud, T. , Joseph, W. , Marozas, V. , Lukosevicius, A. , Ištuk, N. , Šarolić, A. , Lechner, S. , Klonowski, W. , … Széles, J. C. (2019). Current directions in the auricular vagus nerve stimulation I—A physiological perspective. Frontiers in Neuroscience, 13, 854. 10.3389/fnins.2019.00854 31447643 PMC6697069

[brb32343-bib-0026] Kaniusas, E. , Kampusch, S. , Tittgemeyer, M. , Panetsos, F. , Gines, R. F. , Papa, M. , Kiss, A. , Podesser, B. , Cassara, A. M. , Tanghe, E. , Samoudi, A. M. , Tarnaud, T. , Joseph, W. , Marozas, V. , Lukosevicius, A. , Ištuk, N. , Lechner, S. , Klonowski, W. , Varoneckas, G. , … Šarolić, A. (2019). Current directions in the auricular vagus nerve stimulation II—An engineering perspective. Frontiers in Neuroscience, 13, 772. 10.3389/fnins.2019.00772 31396044 PMC6667675

[brb32343-bib-0027] Kiyokawa, J. , Yamaguchi, K. , Okada, R. , Maehara, T. , & Akita, K. (2014). Origin, course and distribution of the nerves to the posterosuperior wall of the external acoustic meatus. Anatomical Science International, 89, 238–245. 10.1007/s12565-014-0231-4 24604237

[brb32343-bib-0028] Lewine, J. D. , Paulson, K. , Bangera, N. , & Simon, B. J. (2019). Exploration of the impact of brief noninvasive vagal nerve stimulation on EEG and event‐related potentials. Neuromodulation, 22, 564–572. 10.1111/ner.12864 30288866

[brb32343-bib-0029] Mercante, B. , Deriu, F. , & Rangon, C. ‐ M. (2018). Auricular neuromodulation: The emerging concept beyond the stimulation of vagus and trigeminal nerves. Medicines, 5, 10. 10.3390/medicines5010010 PMC5874575 29361732

[brb32343-bib-0030] Nasi‐Er, B. G. , Wenhui, Z. , HuaXin, S. , Xianhui, Z. , Yaodong, L. , Yanmei, L. , Hongli, W. , TuEr‐Hong, Z.‐l. , Qina, Z. , & BaoPeng, T. , (2019). Vagus nerve stimulation reduces ventricular arrhythmias and increases ventricular electrical stability. Pacing and Clinical Electrophysiology, 42, 247–256. 30552763 10.1111/pace.13585

[brb32343-bib-0031] Nonis, R. , D'ostilio, K. , Schoenen, J. , & Magis, D. (2017). Evidence of activation of vagal afferents by non‐invasive vagus nerve stimulation: An electrophysiological study in healthy volunteers. Cephalalgia, 37, 1285–1293. 10.1177/0333102417717470 28648089 PMC5680905

[brb32343-bib-0032] Oken, B. S. (1997). Statistics for evoked potential. In K.H. Chiappa (Ed.), Evoked potentials in clinical medicine (3rd ed., pp. 565–77). Lippincot‐Raven.

[brb32343-bib-0033] Peuker, E. T. , & Filler, T. J. (2002) The nerve supply of the human auricle. Clinical Anatomy, 15, 35–37. 10.1002/ca.1089 11835542

[brb32343-bib-0034] Polak, T. , Dresler, T. , Zeller, J. B. M. , Warrings, B. , Scheuerpflug, P. , Fallgatter, A. J. , Deckert, J. , & Metzger, F. G. (2014) Vagus somatosensory evoked potentials are delayed in Alzheimer's disease, but not in major depression. European Archives of Psychiatry and Clinical Neuroscience, 264, 263–267. 10.1007/s00406-013-0415-2 23736883

[brb32343-bib-0035] Polak, T. , Ehlis, A.‐C. , Langer, J. B. M. , Plichta, M. M. , Metzger, F. , Ringel, T. M. , & Fallgatter, A. J. (2007) Non‐invasive measurement of vagus activity in the brainstem—A methodological progress towards earlier diagnosis of dementias? Journal of Neural Transmission, 114, 613–619. 10.1007/s00702-007-0625-8 17308983

[brb32343-bib-0036] Polak, T. , Herrmann, M. J. , Müller, L. D. , Zeller, J. B. M. , Katzorke, A. , Fischer, M. , Spielmann, F. , Weinmann, E. , Hommers, L. , Lauer, M. , Fallgatter, A. J. , & Deckert, J. (2017) Near‐infrared spectroscopy (NIRS) and vagus somatosensory evoked potentials (VSEP) in the early diagnosis of Alzheimer's disease: Rationale, design, methods, and first baseline data of the Vogel study. Journal of Neural Transmission, 124, 1473–1488. 10.1007/s00702-017-1781-0 28864837

[brb32343-bib-0037] Polak, T. , Markulin, F. , Ehlis, A.‐C. , Langer, J. B. M. , Ringel, T. M. , & Fallgatter, A. J. (2009) Far field potentials from brain stem after transcutaneous vagus nerve stimulation: Optimization of stimulation and recording parameters. Journal of Neural Transmission, 116, 1237–1242. 10.1007/s00702-009-0282-1 19728032

[brb32343-bib-0038] Polak, T. , Weise, D. , Metzger, F. , Ehlis, A. C. , Langer, J. B. , Schramm, A. , Fallgatter, A. J. , & Classen, J. (2011) Vagus nerve somatosensory evoked potentials in Parkinson's disease. Journal of Neurology, 258, 2276–2277. 10.1007/s00415-011-6084-z 21559938

[brb32343-bib-0039] Polak, T. , Zeller, D. , Fallgatter, A. J. , & Metzger, F. G. (2013) Vagus somatosensory‐evoked potentials are prolonged in patients with multiple sclerosis with brainstem involvement. NeuroReport, 24, 251–253. 10.1097/WNR.0b013e32835f00a3 23407276

[brb32343-bib-0040] Rong, P. , Liu, A. , Zhang, J. , Wang, Y. , Yang, A. , Li, L. , Ben, H. , Li, L. , Liu, R. , He, W. , Liu, H. , Huang, F. , Li, X. , Wu, P. , & Zhu, B. , (2014) An alternative therapy for drug‐resistant epilepsy: Transcutaneous auricular vagus nerve stimulation. Chinese Medical Journal, 127, 300–304. 24438620

[brb32343-bib-0041] Rong, P.‐J. , Fang, J.‐L. , Wang, L.‐P. , Meng, H. , Liu, J. , Ma, Y.‐G. , Ben, H. , Li, L. , Liu, R.‐P. , Huang, Z.‐X. , Zhao, Y.‐F. , Li, X. , Zhu, B. , & Kong, J. (2012) Transcutaneous vagus nerve stimulation for the treatment of depression: A study protocol for a double blinded randomized clinical trial. BMC Complementary and Alternative Medicine [Electronic Resource], 12, 255. 10.1186/1472-6882-12-255 PMC3537743 23241431

[brb32343-bib-0042] Safi, S. , Ellrich, J. , & Neuhuber, W. (2016) Myelinated axons in the auricular branch of the human vagus nerve. Anatomical Record, 299, 1184–1191. 10.1002/ar.23391 27342906

[brb32343-bib-0043] Shim, H. J. , Kwak, M. Y. , An, Y.‐H. , Kim, D. H. , Kim, Y. J. , & Kim, H. J. (2015) Feasibility and safety of transcutaneous vagus nerve stimulation paired with notched music therapy for the treatment of chronic tinnitus. Journal of Audiology & Otology, 19, 159–167. 10.7874/jao.2015.19.3.159 26771015 PMC4704553

[brb32343-bib-0044] Shiozawa, P. , Da Silva, M. E. , Carvalho, T. C. D. , Cordeiro, Q. , Brunoni, A. R. , & Fregni, F. (2014) Transcutaneous vagus and trigeminal nerve stimulation for neuropsychiatric disorders: A systematic review. Arquivos De Neuro‐Psiquiatria, 72, 542–547. 10.1590/0004-282X20140061 25054988

[brb32343-bib-0045] Silberstein, S. D. , Calhoun, A. H. , Lipton, R. B. , Grosberg, B. M. , Cady, R. K. , Dorlas, S. , Simmons, K. A. , Mullin, C. , Liebler, E. J. , Goadsby, P. J. , & Saper, J. R. (2016) Chronic migraine headache prevention with noninvasive vagus nerve stimulation: The EVENT study. Neurology, 87, 529–538. 10.1212/WNL.0000000000002918 27412146 PMC4970666

[brb32343-bib-0046] Skraus, T. , Kiess, O. , Hösl, K. , Terekhin, P. , Kornhuber, J. , & Forster, C. (2013) CNS BOLD fMRI effects of sham‐controlled transcutaneous electrical nerve stimulation in the left outer auditory canal—A pilot study. Brain Stimulation, 6, 798–804. 23453934 10.1016/j.brs.2013.01.011

[brb32343-bib-0047] Staats, P. , Giannakopoulos, G. , Blake, J. , Liebler, E. , & Levy, R. M. (2020). The use of non‐invasive vagus nerve stimulation to treat respiratory symptoms associated with COVID‐19: A theoretical hypothesis and early clinical experience. Neuromodulation, 23(6), 784–788. 32342609 10.1111/ner.13172 PMC7267613

[brb32343-bib-0048] Stefan, H. , Kreiselmeyer, G. , Kerling, F. , Kurzbuch, K. , Rauch, C. , Heers, M. , Kasper, B. S. , Hammen, T. , Rzonsa, M. , Pauli, E. , Ellrich, J. , Graf, W. , & Hopfengärtner, R. (2012) Transcutaneous vagus nerve stimulation (t‐VNS) in pharmacoresistant epilepsies: A proof of concept trial. Epilepsia, 53, e115–e118. 10.1111/j.1528-1167.2012.03492.x 22554199

[brb32343-bib-0049] Suk, W. C. , Kim, S. J. , Chang, D. S. , & Lee, H. Y. (2018) Characteristics of stimulus intensity in transcutaneous vagus nerve stimulation for chronic tinnitus. Journal of International Advanced Otology, 14, 267–272. 10.5152/iao.2018.3977 PMC6354472 30256201

[brb32343-bib-0050] Tekdemir, I. , Aslan, A. , & Elhan, (1998) A clinico‐anatomic study of the auricular branch of the vagus nerve and Arnold's ear‐cough reflex. Surgical and Radiologic Anatomy, 20, 253–257. 9787391

[brb32343-bib-0051] Trevizol, A. , Barros, M. D. , Liquidato, B. , Cordeiro, Q. , & Shiozawa, P. (2015) Vagus nerve stimulation in neuropsychiatry: Targeting anatomy‐based stimulation sites. Epilepsy & Behavior, 51, 18. 10.1016/j.yebeh.2015.07.009 26262931

[brb32343-bib-0052] Usami, K. , Kawai, K. , Sonoo, M. , & Saito, N. (2013) Scalp‐recorded evoked potentials as a marker for afferent nerve impulse in clinical vagus nerve stimulation. Brain Stimulation, 6, 615–623. 10.1016/j.brs.2012.09.007 23088852

[brb32343-bib-0053] Ventureyra, E. C. G. (2000) Transcutaneous vagus nerve stimulation for partial onset seizure therapy. A new concept. Childs Nervous System, 16, 101–102. 10.1007/s003810050021 10663816

[brb32343-bib-0054] Yakunina, N. , Kim, S. S. , & Nam, E. ‐ C. (2017) Optimization of transcutaneous vagus nerve stimulation using functional MRI. Neuromodulation, 20, 290–300. 10.1111/ner.12541 27898202

[brb32343-bib-0055] Yuan, H. , & Silberstein, S. D. (2016) Vagus nerve and vagus nerve stimulation, a comprehensive review: Part I. Headache, 56, 71–78. 10.1111/head.12647 26364692

[brb32343-bib-0056] Yuan, T.‐F. , Li, A. , Sun, X. , Arias‐Carrión, O. , & Machado, S. (2016) Vagus nerve stimulation in treating depression: A tale of two stories. Current Molecular Medicine, 16, 33–39. 10.2174/1566524016666151222143609 26695696

